# Myelofibrosis: molecular and cell biological aspects

**DOI:** 10.1186/1755-1536-5-S1-S21

**Published:** 2012-06-06

**Authors:** Hans Kreipe, Guntram Büsche, Oliver Bock, Kais Hussein

**Affiliations:** 1 Institute of Pathology, Hannover Medical School, Hannover, Germany

## Abstract

A subset of myeloproliferative disorders (MPN) and myelodyplastic syndromes (MDS) evolves to fibrosis of the bone marrow associated with haematopoietic insufficiency. We have been interested in chemokines involved in fibrogenesis within the bone marrow. Besides TGFβ we could identify a number of additional mediators including osteoprotegerin and bone morphogenic proteins. In MPN JAK2 or MPL mutation are not linked to the propensity for bone marrow fibrosis. The hypothesis that an increased intramedullary decay of megakaryocytes undergoing appotosis takes place within the marrow, thus liberating fibrogenic cytokines, could not be confirmed. On the contrary, megakaryocytes in primary fibrosis revealed low expression of proapoptotic genes such as BNIP3. Interestingly, BNIP 3 expression was down regulated in megakaryocytic cell lines kept in hypoxic conditions. Furthermore, expression arrays revealed hypoxia inducible genes to be up-regulated in primary myelofibrosis. Fibrotic MPN are characterized by aberrant proplatelet formation which represent cytoplasmic pseudopodia and normally extend into the sinus. In fibrotic MPN orientation of proplatelet growth appears to be disturbed, which could lead to an aberrant deposition of platelets in the marrow with consecutive liberation of fibrogenic cytokines.

## Introduction

Myeloproliferative Neoplasms (MPN) represent clonal proliferations of pathological haematopoietic stem cell, which have become independent from physiological growth control but retain the ability to differentiate completely into all hematopoietic lineages. All or a subset of the haematopoietic lineages can be affected, resulting in a mono-, bi- or trilinear proliferation of megakaryocytic, erythroid and granulocytic precursor cells. MPN encompass chronic myeloid leukaemia (CML), polycythemia vera (PV), essential thrombocythemia (ET) and primary myelofibrosis (PMF). Chronic neutrophilic leukaemia (CNL) and chronic eosinophilic leukaemia (CEL) represent rare forms of MPN [1]. Although stable for a long period from years to decades all MPN may progress to either blast crisis or to bone marrow fibrosis. The propensity, however, to develop these typical complications varies considerably between the subtypes. Although the WHO classification suggests that the fibrotic potential of MPN with predominant thrombocytosis such as ET and cellular PMF can be differentiated on the ground of morphology alone, the few prospective clinical trials which have been performed in the field have suggested that this concept has to be questioned [2].

Therefore, molecular markers derived from a deeper understanding of pathogenesis are necessary to achieve a more accurate and reproducible classification of MPN according to their risk of fibrotic progression.

## Results and discussion

### Chemokines involved in Fibrogenesis

Transforming growth factor beta-1 (TGF beta-1) is a potent inducer of fibrosis and has been shown to be essential for the development of bone marrow fibrosis in an animal model of PMF [3]. Megakaryocytes and platelets have been suggested as the major cellular source of TGF beta-1 in PMF. We analysed total bone marrow cells from bone marrow trephines by PCR of cDNA, and found TGF beta-1 mRNA expression to be increased in some but not all cases of IMF (n = 21), with highest values in fibrotic cases [4]. Unexpectedly, increased values were also observed in essential thrombocythaemia (ET, n = 11) when compared to non-neoplastic haematopoiesis (n = 38). Megakaryocytes isolated by laser microdissection displayed elevated TGF beta-1 mRNA levels in most of the MPN samples with no significant differences discernible between fibrotic IMF, polycythaemia vera (PV) and ET. TGF beta-1 protein was predominantly expressed by the myeloid lineage in Ph-negative MPN and non-neoplastic haematopoiesis, which, however, displayed lower expression. Thus, enhanced TGF beta-1 expression occurs in megakaryocytes as well as myeloid cells in Ph-negative MPN. TGF beta-1 may be necessary, but is not sufficient, to induce bone marrow fibrosis in PMF because non-fibrotic Ph-negative MPN entities share this feature with PMF and cannot be discriminated from each other on the basis of TGF beta-1 expression. Among the cytokines which we found to be increased in fibrotic MPN were PDGF [5] and FGF [6] but a strict correlation to fibrosis could not be established in either case because also ET demonstrated overexpression.

Advanced myelofibrosis with osteosclerosis and increase and thickening of bone trabeculae is typically contrasted by the absence or sparse presence of osteoclasts. Because osteoclast formation can be inhibited by osteoprotegerin (OPG) we investigated OPG expression in PMF with severe fibrosis and osteosclerosis, which expressed significantly higher (up to 71-fold) OPG mRNA levels when compared with prefibrotic cellular PMF and control cases. The receptor activator of nuclear factor kappaB ligand (RANKL), a positive regulator of osteoclast differentiation and putative antagonist of OPG was overexpressed by up to 34-fold exclusively in advanced PMF. Case-specific calculation of the RANKL/OPG ratio in advanced PMF showed a wide range without significant differences when compared with the prefibrotic PMF and non-neoplastic haematopoiesis. Immunohistochemical detection of OPG protein revealed strong labelling of endothelial cells within proliferating vessels in fibrotic PMF and heterogeneously labelled megakaryocytes, and fibroblasts. Osteosclerosis and impaired osteoclast function in PMF appears to be associated with upregulated endothelial OPG expression but concomitant reduction of the antagonist RANKL could not be demonstrated. It appears, that osteosclerosis in PMF is associated with increased endothelial OPG expression without concomitant RANKL downregulation [7].

The involvement of members of the bone morphogenetic protein (BMP) family in aberrant bone marrow matrix homeostasis in PMF has not yet been investigated. Therefore, we analyzed expression of BMP1, an activator of latent transforming growth factor beta-1 (TGFbeta-1) and processor of collagen precursors, and other BMPs in bone marrow from PMF patients and controls (n = 95). Expression of BMP1, BMP6, BMP7, and BMP-receptor 2 was significantly increased in advanced stages of myelofibrosis compared with controls (P < or = 0.01), and enhanced levels of BMP6 expression were already evident in prefibrotic stages of PMF. Immunohistochemistry showed that bone marrow stromal cells and megakaryocytes were the major cellular sources of BMP1 protein. Because TGFbeta-1 and basic fibroblast growth factor have been shown to be important in the development of myelofibrosis, we studied the induction of BMPs by these cytokines in cultured fibroblasts. Fibroblasts treated with TGFbeta-1 showed a pronounced up-regulation of BMP6, suggesting that stromal cells may be susceptible to BMP activation by cytokines with a proven role in the pathogenesis of PMF. We conclude that BMP family members may play an important role in the pathogenesis of myelofibrosis in PMF and are apparently induced by cytokines such as TGFbeta-1 [8].

### Role of JAK2 and MPL

Although the cellular phase of PMF has been demonstrated to represent a clonal disease [9], it shares with all other Ph- MPN the peculiar phenomenon that only a proportion of cells is affected by JAK2 or MPL mutation [10]. We have been interested whether in follow-up biopsies from MPN patient an increase in allele burden was associated with increased fiber deposition and whether ET differs from cellular PMF. Histopathology of 490 MPN cases was correlated with the allelic burden of JAK2(V617F) and MPL(W515L) [11]. Ph-negative MPN entities largely overlap with regard to JAK2(V617F) and MPL(W515L) allele burden, but ET displayed mutant allele burden <50%. PMF with different stages of myelofibrosis all yielded similar JAK2(V617F) allele burden. At initial presentation one-quarter of prefibrotic PMF cases exhibited an allele burden exceeding 50% (38% median JAK2(V617F) alleles, n = 102). In ET, its main differential diagnosis, not a single case was found with >40% JAK2(V617F) alleles (median, 24% JAK2(V617F) alleles; n = 90; p < 0.001). Increase in JAK2(V617F) alleles during follow-up could not be linked to fibrosis or blastic progression but was related to polycythemic transformation in ET. MPL(W515L) was found in 3% of ET and 8% of PMF, with a significantly higher percentage of mutated alleles in fibrotic than prefibrotic PMF (median, 78% MPL(W515L) alleles; p < 0.05). Consequently, typical histological findings in ET and prefibrotic PMF correlate with significant differences in mutant allelic burden of JAK2(V617F), but not of MPL(W515L). By contrast to JAK2(V617F), MPL (W515L) shows a higher percentage of mutated alleles in fibrotic than in prefibrotic cases. In Ph-negative MPN with the differential diagnosis between ET and prefibrotic PMF, a JAK2(V617F) allele burden >50% favors a diagnosis of prefibrotic PMF. This does, however, not imply that JAK2 allele burden is linked to development of fibrosis in individual cases, because allelic burden stayed stable during progression [12].

In a next step we evaluated whether differences between JAK2 mutated and unmutated MPN cases with fibrosis could be established. Because the JAK-STAT signaling pathway is involved in the regulation of genes encoding matrix metalloproteinases (MMPs) and bone marrow fibrosis most likely represents an imbalance between synthesis and turnover of collagen fibers, we hypothesized that differences exist between mutated and unmutated cases. The expression of MMPs, their tissue inhibitors (TIMPs), and collagen types in relation to the JAK2 status (V617F mutation versus wild-type) were studied in cellular PMF (n = 64). Whereas no correlation was found between the JAK2 status and MMP gene products, there was an evident association with the stage of disease. Membrane type 1-MMP (MMP-14) was overexpressed by up to 80-fold in advanced stages that progressed to fibrosis (P < 0.001), and megakaryocytes and endothelial cells were unmasked as the major cellular source. By contrast, a significantly higher expression of neutrophil collagenase (MMP-8) was encountered in the prefibrotic stages of cIMF (P < 0.001). Altogether, the stepwise progress of myelofibrosis in cIMF was associated with expression of a defined subset of target genes as shown in sequential trephine biopsies of cIMF patients. Obviously, the expression of matrix-modeling genes in cIMF is not influenced by the JAK2 mutation status but is predominantly related to the stage of disease [12].

### Intramedullary apoptosis of megakaryocytes

It has been speculated that an increase of megakaryocytic decay and apoptosis in the bone marrow of MPN may lead to an enhanced liberation of cytokines with a fibrogenic potential [13]. In order to identify factors involved in the aberrantly regulated apoptosis of megakaryocytes in primary myelofibrosis (PMF), the mRNA expression of human megakaryocytes in the bone marrow of PMF was quantified by real-time polymerase chain reaction low-density arrays. The mRNA from 200 to 300 laser-microdissected megakaryocytes per case from PMF (n = 22) and control (n = 10) bone marrow was reverse-transcribed into cDNA by random priming and subsequently amplified by primer-specific cDNA amplification. The mRNA of corresponding total bone marrow cells was reverse-transcribed into cDNA without the following amplification. For relative mRNA quantification, custom-made TaqMan low-density arrays with a setup of 48 different genes were applied. In addition, methylation analysis and immunohistochemistry of a selected candidate gene were accomplished. A trend toward an overall downregulation of apoptosis-associated genes could be observed in megakaryocytes, whereas the total bone marrow cellularity exhibited an overall upregulation of these factors. Among several candidates with statistically significant deregulation BCL2/adenovirus E1B 19 kDa interacting protein 3 (BNIP3) and protein kinase C beta1 were shown to be the most aberrantly expressed genes. Apoptosis-related gene expression profiling of human megakaryocytes reveals a set of candidates, most notably BNIP3, indicating that the increase of megakaryocytes in myeloproliferative neoplasia might not only be the result of increased proliferation but also of disturbed apoptosis [14]. Thus megakaryocytes in PMF exhibit rather a decrease of apoptosis than an increase and an exaggerated apoptotic decay as a source of fibrogenic cytokines does not take place.

### Potential role of hypoxia

When studying gene expression in PMF we encountered an increased expression of genes related to hypoxia. We investigated bone marrow core biopsies in PMF showing various degrees of myelofibrosis by custom-made low density arrays (LDA) representing target genes with designated roles in synthesis of extracellular matrix, matrix remodelling, cellular adhesion and motility. Among a set of 11 genes up-regulated in advanced stages of PMF (P < or = 0.01) three candidates, PTK2 protein tyrosine kinase 2 (PTK2), transforming growth factor beta type II receptor (TGFBR2) and motility-related protein-1 (CD9 molecule, CD9), were investigated in more detail. PTK2, TGFBR2 and CD9 were significantly overexpressed in larger series of advanced PMF stages (P < or = 0.01 respectively). Endothelial cells of the increased microvessel network in PMF could be identified as a predominant source for PTK2, TGFBR2 and CD9. CD9 also strongly identified activated fibroblasts in advanced myelofibrosis [15]. As CD9 expression is induced by hypoxia and angiogenesis is a prominent feature in PMF hypoxia emerges as a potential regulator of bone marrow fibrosis.

### Increased proplatelet formation in PMF

Although megakaryocytes have been identified as the source of thrombocytes more than 100 years ago there is still no consensus for how platelets are assembled. Besides the cytoplasmic fragmentation model there is the proplatelet model which is based on long cytoplasmic extensions observed in megakaryocytes in vivo [3] and in vitro. Pseudopodial cytoplasmic extensions of megakaryocytes according to this model provide the essential intermediate structure for the generation of platelets. The cytoplasmic extensions can reach 500 μm in length and are presumed to get into contact with sinuses for the release of platelets into the blood stream [16]. Proplatelets are usually not obvious in conventional bone marrow histology and they may escape immunohistochemical detection by the most frequently used megakaryocytic markers factor VIII-related antigen and CD61. We have visualized proplatelet formation in MPN in situ by immunohistochemical detection of glycoprotein Ib (platelet) alpha polypeptide (GP1BA; CD42). The expression of GPIX, heterodimer complex with GP1B and GPV, in the platelet membrane characterized an early differentiation. Immunohistochemical stainings were evaluated by conventional microscopy and confocal microscopy. In trephines from essential thrombocythaemia (ET, n = 10), fibrotic (n = 10) and prefibrotic (n = 10) primary myelofibrosis (PMF) there was a significant increase of proplatelet density compared with normal bone marrow samples (n = 10; p < 0.001). Manifest fibrosis exhibited the highest density and volume ratio with significant differences to non-fibrotic PMF (p < 0.001) and ET (p < 0.001). We could demonstrate that besides megakaryocytic proliferation extensive pseudopodial proplatelet formation provides a hallmark of MPN. Obviously fibrosing differ from non-fibrosing MPN by density and size of aberrant proplatelets [17].

The exaggerated proplatelet formation appears to be associated with an increased production of thrombospodin. Thrombospondins (TSP) are factors sharing pro-fibrotic and anti-angiogenic properties, which have yet not been studied in PMF before. We investigated the expression of TSP-1 and TSP-2 in PMF related to the stage of myelofibrosis (n = 51) and in individual follow-up biopsies by real-time PCR, immunohistochemistry, and confocal laser scanning microscopy (CLSM). TSP-1 was significantly overexpressed (p < 0.05) in all stages of PMF when compared to controls. Individual follow-up biopsies showed involvement of TSP-1 during progressive myelofibrosis. TSP-2 was barely detectable but 40% of cases with advanced myelofibrosis showed a strong expression. Megakaryocytes and interstitial proplatelet formations were shown to be the relevant source for TSP-1 in PMF. Stromal cells like endothelial cells and fibroblasts showed no TSP-1 labeling when double-immunofluorescence staining and CLSM were applied. Based on its dual function, TSP-1 in PMF is likely to be a mediator within a pro-fibrotic environment which discriminates PMF from ET cases. On the other hand, TSP-1 is a factor acting (ineffectively) against exaggerated angiogenesis. Both features suggest TSP-1 to be a biomarker for monitoring a PMF-targeted therapy. The higher number of proplatelets in PMF is most likely responsible for the increase of thrombospodin expression in comparison to ET.

## Competing interests

The authors declare that they have no competing interests.

**Figure 1:**
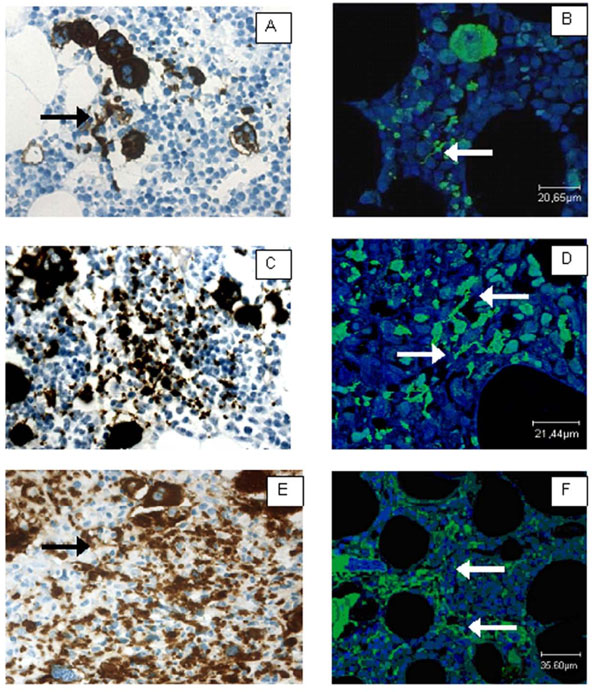
**A-F: Immunohistochemistry for CD42b in ET (A, B), prefibrotic PMF mf0 (C, D) and fibrotic PMF (E, F)**. Confocal laser microscopy reveals dendritic processes (B, D, F; green staining) whereas in conventional immunohistochemistry predominantly cross sections are seen (brown staining) which increase in density in fibrotic PMF (CD42b; ×400) [17].
